# Therapeutic drug monitoring of linezolid in Chinese premature neonates: a population pharmacokinetic analysis and dosage optimization

**DOI:** 10.1128/aac.01148-24

**Published:** 2024-10-09

**Authors:** Lu-fen Duan, Jing-jing Li, Li-rong Shen, Xiang-long Chen, Yan-xia Yu, Zu-ming Yang, Qian Zhang, Yan Cai, Jia-hui Li, Juan Wu, Han-zhen Zhao, Jin-hui Xu, Zong-tai Feng, Lian Tang

**Affiliations:** 1 Department of Pharmacy, The Affiliated Suzhou Hospital of Nanjing Medical University, Suzhou Municipal Hospital, Suzhou, Jiangsu, China; 2 Neonatology Department, The Affiliated Suzhou Hospital of Nanjing Medical University, Suzhou Municipal Hospital, Suzhou, Jiangsu, China; 3 Gusu School, Nanjing Medical University, Suzhou, China; Providence Portland Medical Center, Portland, Oregon, USA

**Keywords:** linezolid, premature neonates, PPK, TDM, dosage optimization

## Abstract

This study aimed to develop a pharmacokinetic model of linezolid in premature neonates and evaluate and optimize the administration regimen. Liquid chromatography-tandem mass spectrometry (LC-MS/MS) was used to detect the blood concentration data of 54 premature neonates after intravenous administration of linezolid, and the relevant clinical data were collected. The population pharmacokinetic (PPK) model was established by nonlinear mixed effects modeling. Based on the final model parameters, the optimal administration regimen of linezolid in premature neonates with different body surface areas (BSA) was simulated and evaluated. The pharmacokinetic properties of linezolid in premature neonates are best described by a single-compartment model with primary elimination. The population typical values for apparent volume of distribution and clearance were 0.783 L and 0.154 L/h, respectively. BSA was a statistically significant covariate with clearance (CL) and volume of distribution (V_d_). Monte Carlo simulations showed that the optimal administration regimen for linezolid in premature neonates was 6 mg/kg q8h for BSA 0.11 m^2^, 7 mg/kg q8h for BSA 0.13 m^2^, and 9 mg/kg q8h for BSA 0.15 m^2^ with minimum inhibitory concentration (MIC) ≤1 mg/L, 7 mg/kg q8h for BSA 0.11 m^2^, 8 mg/kg q8h for BSA 0.13 m^2^, and 10 mg/kg q8h for BSA 0.15 m^2^ with MIC = 2 mg/L. A pharmacokinetic model was developed to predict the blood concentration on linezolid in premature neonates. Based on this model, the optimal administration regimen of linezolid in premature neonates needs to be individualized according to different BSA levels.

## INTRODUCTION

Linezolid is a synthetic antibacterial drug of the oxazolidinone class; it can be used in the treatment of neonatal sepsis or pneumonia caused by methicillin-resistant coagulase-negative *Staphylococcus* (MRCoNS) or methicillin-resistant *Staphylococcus aureus* (MRSA) (1).

Linezolid requires therapeutic drug monitoring (TDM) due to differences in linezolid exposure between individuals are related to differences in efficacy and adverse reactions (2). Linezolid-induced thrombocytopenia (LIT) is the main factor limiting the clinical application of linezolid. Our previous research results showed that LIT occurred in about one-fifth of neonates treated with linezolid (3). At present, there are relatively few studies on TDM and pharmacokinetic/pharmacodynamic (PK/PD) of linezolid in neonates, and these studies still have certain limitations. Sicard et al. performed a retrospective study of linezolid pharmacokinetics in 16 premature infants with 24 linezolid plasma concentrations. There were some limitations in this study, such as a small sample size and two administration methods of linezolid (intravenous and oral) (4). Thibault et al. retrospectively studied data from 26 infants, collected 78 linezolid plasma concentrations, and developed a one-compartment model of linezolid. The final model included postnatal age (PNA), weight on clearance, and weight on volume of distribution. However, there were also some limitations in this study, such as the small sample size and the inclusion of infants who were not all neonates (5).

Our previous research results showed that there were significant individual differences in the initial linezolid serum trough concentration (C_min_) in neonatal patients，with a compliance rate as low as 32.26%, 51.60% of neonates had higher C_min_ than the target range (6). This may be related to the fact that (i) most of the neonates included in this study were premature neonates, and the renal excretion capacity of premature neonates was worse than that of full-term neonates; and (ii) the recommended dosage for premature neonates older than 7 days after birth was the same as that for full-term neonates in the drug introduction, which may be high for premature neonates. The purpose of our study was to develop a pharmacokinetic model of premature neonates, which could be used to realize individualized medication of linezolid in premature neonates.

## RESULTS

### Demographics and characteristics of patients

A total of 54 infants, 32 males and 22 females, were included in the study. The demographic and clinical characteristics of the study population are presented in Table 1. The medication of linezolid therapy and clinical outcomes of the premature infants are shown in Table 2.

**TABLE 1:** Demographic and clinical characteristics of the study population

Characteristic	Value (*n* = 54)
Demographics	
Gestational age (wk), mean ± SD	31.00 ± 2.74
Postmenstrual age (wk), mean ± SD	33.16 ± 2.77
Postnatal age (d), median [IQR]	13.00 (8.00,19.25)
Birth weight (g), mean ± SD	1449.44 ± 496.73
Current weight (g), mean ± SD	1571.30 ± 503.81
Gender (male), *n* (%)	32(59.26)
BSA (m^2^)	0.13 ± 0.03
Apgar score at 1 min, median [IQR]	8.00 (7.00, 9.00)
Apgar score at 5 min, median [IQR]	9.00 (8.00, 9.25)
Infectious disease, *n* (%)	
Pneumonia	53 (98.15)
Bloodstream infection	48 (88.89)
Necrotizing enterocolitis	5(9.26)
Suppurative meningitis	1 (1.85)
Complications, *n* (%)	
Respiratory failure	42 (77.78)
Respiratory distress syndrome	29 (53.70)
Asphyxia	15 (27.78)
Septic shock	6 (11.11)
Pathogen, *n* (%)	31 (57.41)
Coagulase-negative *Staphylococcus*	27
*Enterococcus faecium*	3
*Staphylococcus aureus*	1
Linezolid MIC values	
1 mg/L	14 (45.16)
2 mg/L	17 (54.84)
Laboratory values at baseline	
Hemoglobin (g/L), mean ± SD	126.43 ± 20.90
Platelet (10^9^/L), mean ± SD	197.65 ± 95.16
Total bilirubin (μmol/L), median [IQR]	81.36 (40.93, 136.94)
Albumin (g/L), median [IQR]	31.95 (29.33, 34.43)
ALT(U/L), median [IQR]	9.00 (5.75, 17.00)
Cr (μmol/L), mean ± SD	40.55 ± 12.96
CLcr (mL/min/1.73 m^2^), median [IQR]	29.46 (24.79, 40.41)

**TABLE 2:** The medication of linezolid therapy and clinical outcomes of premature infants

Characteristic	Value (*n* = 54)
Linezolid medication	
Medication days (d), median [IQR]	10.00 (9.00, 11.00)
Concomitant antibiotics, *n* (%)	23 (42.59)
Meropenem	17
Piperacillin-tazobactam	3
Cefoperazone-sulbactam	2
Imipenem-cilastatin	1

### Development of the population pharmacokinetics model

**Fig 1:**
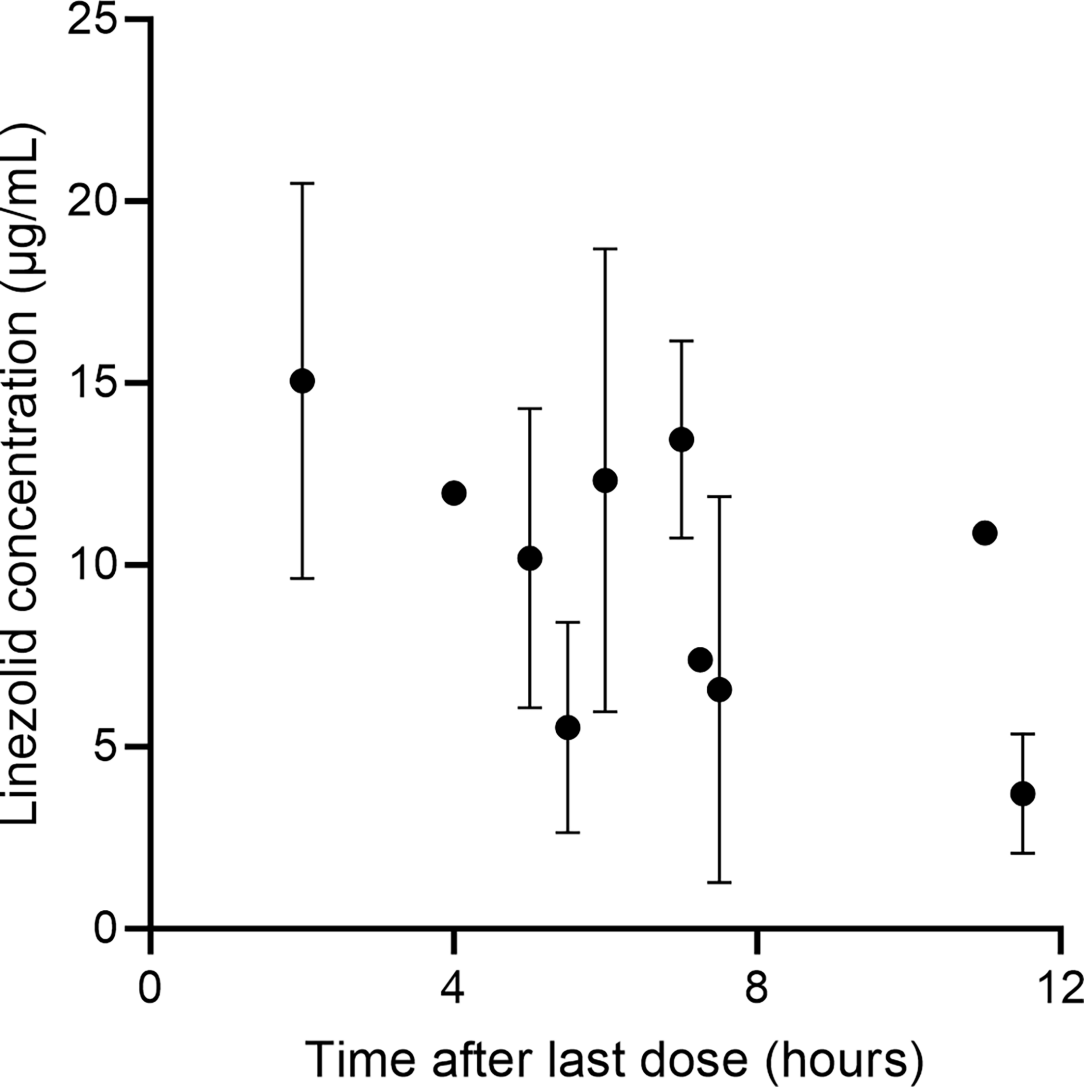
Linezolid concentrations (mean ± SD) vs time.

We collected 84 blood samples. The concentration–time profile of linezolid for the included patients is shown in Fig. 1. The concentration value in the figure is the mean ± SD.

**Fig 2:**
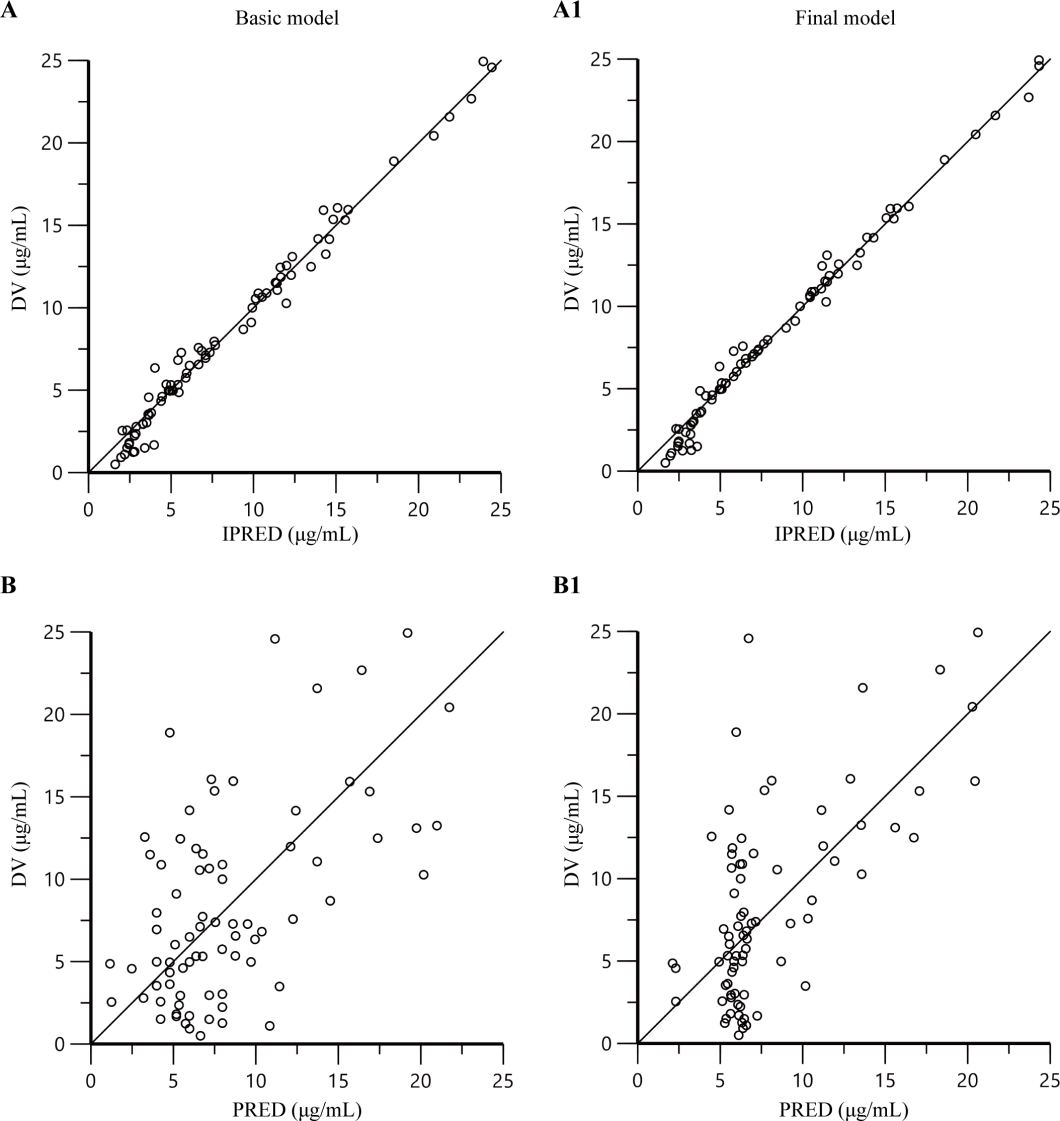
Goodness-of-fit plots for the basic model (**A and B**) and the final model (**A1 and B1**). (**A and A1**) The observed concentrations (DV) versus individual-predicted concentrations (IPRED); (**B and B1**) the DV versus population-predicted concentrations (PRED).

PK characteristics of linezolid were adequately described by a one-compartment model with first-order elimination. The inter-individual variability was described by an exponential random-effects model, and residual variability was fitted with a proportional residual error model. During covariate selection, BSA was the significantly effective covariates for the total body clearance (CL) of linezolid, and BSA was also the significantly effective covariate for the volume of distribution (V). Other covariates were systematically analyzed but were not found to be statistically significant predictors of PK parameters. V with BSA, and CL with BSA most significantly improved the fitness of the model (ΔOFV = 19.84, *P* < 0.001, respectively, Table 3; Fig. 2).

**TABLE 3:** Covariate hypothesis testing in the nodel development^*a*^

Model description	OFV	ΔOFV	*P* value
Basic model	410.89		
Full covariate model(CL-BSA, V-BSA)	391.05		
Backward elimination			
Removing CL-BSA	408.24	17.19	< 0.01
Removing V-BSA	400.77	9.72	< 0.01

^*a*^ OFV, objective function value; V, the volume of distribution; CL, clearance; BSA, body surface area; ΔOFV, change in the OFV compared with reference model.

The final population PK model was as follows:


(1) $$V(L)=0.783\times (BSA/0.127{)}^{1.066}\times exp(\eta V_{d})$$

(2) $$CL\;(L/h)=0.154\times (BSA/0.127{)}^{1.186}\times exp(\eta CL)$$

The population parameters of V and CL estimated in the final model were 0.783 L and 0.154 liters/h (equivalent to CL as 0.098 L/h /kg), respectively. The details are displayed in Table 4.

**TABLE 4:** Population PK parameter estimates in the final model and bootstrap^*a*^^,^^*b*^

Parameter (unit)	Full model			Bootstrap	
	Estimate (shrinkage %)	CV%	95% CI	Median	95% CI
Structural model parameters					
tvV(L)	0.783	4.749	0.709–0.857	0.787	0.712–0.869
θ_BSA-V_ (m^2^)	1.066	30.750	0.413–1.720	1.100	0.311–1.915
tvCL (L/h)	0.154	5.010	0.139–0.169	0.154	0.140–0.172
θ_BSA-CL_ (m^2^)	1.185	21.774	0.671–1.700	1.212	0.555–1.771
Inter-individual variability					
ω^2^CL (%)	0.132 (5.838)	18.58	0.0914–0.172	0.129	0.0886–0.170
Residual variability					
stdev0	1.120	13.183	0.826–1.415	1.052	0.635–1.341

^*a*^ TV, typical population value; CV, coefficient of variation; θ_BSA-V_ is the adjusting factor of the BSA on the V; θ_BSA-CL_ is the adjusting factor of the BSA on the CL; 95% CI, 2.5^th^ and 97.5^th^ percentile of the ranked bootstrap parameter estimate; V, volume of distribution; CL, clearance; ω inter-individual variation; stdev0, standard deviation.

^*b*^ Typical population for a 1.571 Kg neonate.

### Model validation

**Fig 3:**
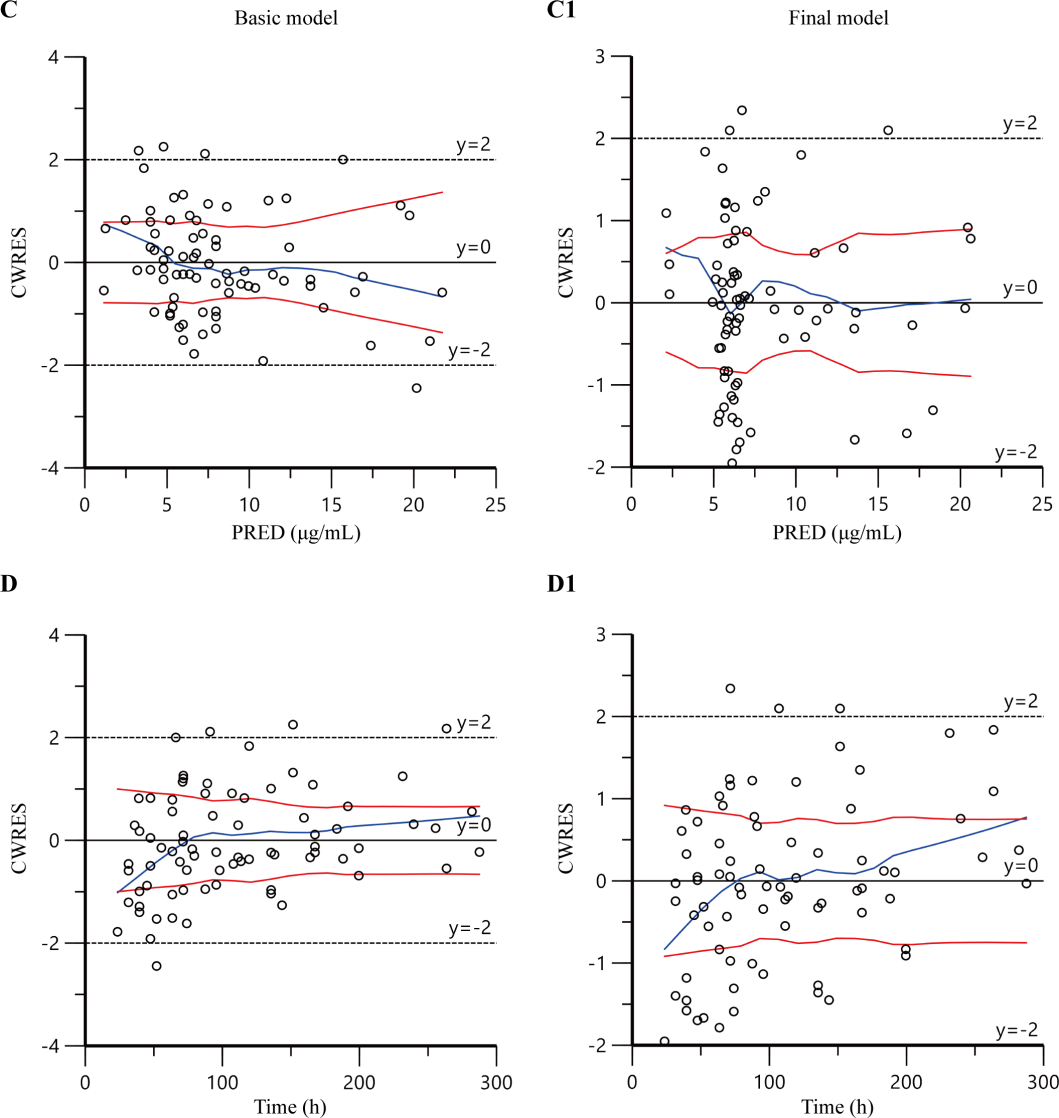
Goodness-of-fit plots for the basic model (C and D) and the final model (C1 and D1). (C and C1) The conditional weighted residuals versus population-predicted concentrations; (D and D1) the conditional weighted residuals versus time.

The diagnostic goodness-of-fit plots obtained from the basic and final PPK model are presented in Fig. 2 and 3. The plots of PRED (B1) and IPRED (A1) versus DV showed no structural deviations in terms of visual biases, and the fit of the final model was improved compared with the basic model. In the plots of conditional weighted residuals (CWRES) versus PRED and time, most concentration data were randomly distributed around 0 and within −2 to +2, which indicated no significant systematic deviations in the model fit. The final model (C1 and D1) showed an improvement in fit over that of the basic model (C and D).

The obtained medians and 95% confidence intervals (CIs) of parameter estimates from a 1,000-run bootstrap sets analysis are shown in Table 4. The parameter estimates from the original data lay within the 95% CIs resulting from the nonparametric bootstrap method, and the 95% CIs did not include zero. The biases between the final model estimates and the bootstrapped median parameter estimates were less than 10% for all parameters, suggesting good stability and robustness of the final model.

**Fig 4:**
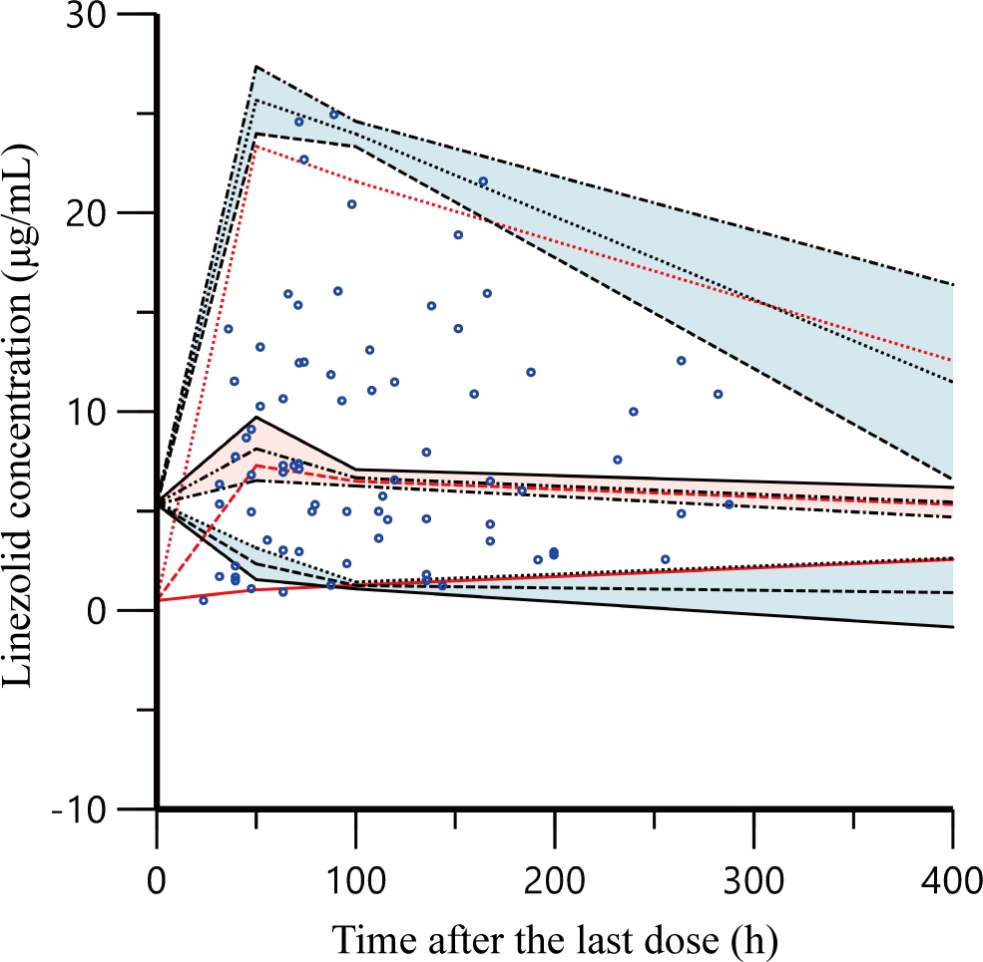
Prediction corrected-visual predictive check of the final model. The observed linezolid concentrations are shown as blue circles. Red solid and dashed lines represent the 5th, 50th, and 95th percentiles of the observed concentrations; the three shaded areas represent the 90% CIs of the 5th, 50th, and 95th percentiles of the simulated concentrations.

Figure 4 shows a prediction corrected-visual predictive check (pc-VPC) of concentration versus time after the last dose. Most of the observed 5th, 50th, and 95th quantiles were within the 90% CIs of the predicted corresponding quantiles, indicating an acceptable consistency between the observed and simulated concentrations. Overall, the evaluation of the linezolid PPK model demonstrated that the final model provided a sufficient description of the data.

### Monte Carlo simulations

A dosing regimen with a dose range of 5–12 mg/kg and dosing interval of 8–12 h based on gestational age resulted in a high rate of success for C_min_ and AUC_0-24h_ after 2 days of administration (Table 5).

**TABLE 5:** Summary of optimal dosage regimen simulation under different BSA levels

BSA (m^2^)	Dosage	Interval	% of patients by distribution of C_min,ss_ (μg/mL)			% of patients by distribution of AUC_0-24_ (mg·h/L)		
			<2 µg/mL	2–8	>8	≤ 80	80–300	≥ 300
0.11	5 mg/kg	q8h	22.5	70.3	7.2	1.8	88.4	9.8
	6 mg/kg	q8h	15.7	71.8	12.5	0.4	89.1	10.5
	7 mg/kg	q8h	11.3	70.1	18.6	0.2	70.3	29.5
	10 mg/kg	q12h	35.9	56.4	7.7	0.3	75.0	24.7
	11 mg/kg	q12h	31.6	59.3	9.1	0.2	65.1	34.7
	12 mg/kg	q12h	29.5	58.8	11.7	0	55.7	44.3
0.13	6 mg/kg	q8h	24.0	69.8	6.2	2.3	88.1	9.6
	7 mg/kg	q8h	18.4	71.2	10.4	0.5	88.9	10.6
	8 mg/kg	q8h	14.5	69.6	15.9	0.3	77.2	22.5
	13 mg/kg	q12h	35.1	56.7	8.2	0.2	68.4	31.4
	14 mg/kg	q12h	31.3	58.5	10.2	0.1	60.4	39.5
	15 mg/kg	q12h	29.9	58.3	11.8	0	52.9	47.1
0.15	8 mg/kg	q8h	20.8	70.1	9.1	0.7	88.9	10.4
	9 mg/kg	q8h	16.5	70.5	13.0	0.3	89.0	10.7
	10 mg/kg	q8h	13.0	69.8	17.2	0.2	71.5	28.3
	17 mg/kg	q12h	31.6	58.1	10.3	0	58.0	42.0
	18 mg/kg	q12h	30.0	58.2	11.8	0	51.6	48.4
	19 mg/kg	q12h	29.1	57.5	13.4	0	46.0	54.0

**Fig 5:**
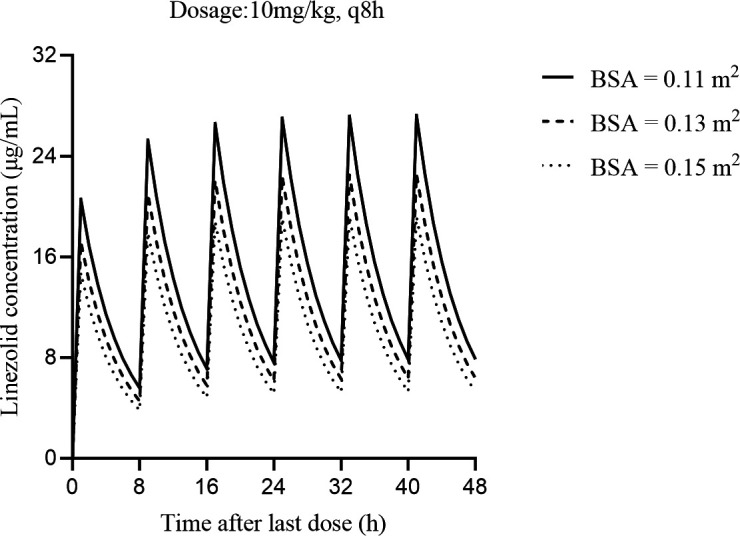
Model-based prediction of linezolid concentrations versus time. Dosage regimen: 10 mg/kg, q8h; BSA = 0.11, 0.13, 0.15 m^2^.

**Fig 6:**
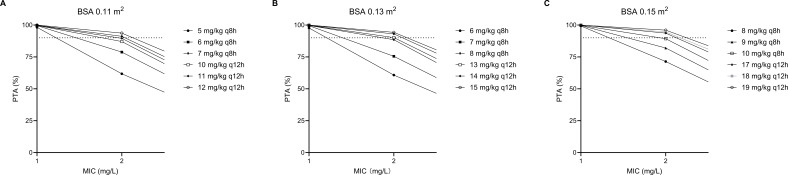
Target attainment rates by MIC. MIC, minimum inhibitory concentration (mg/L); PTA, probability of target attainment (%) dotted line represents 90% PTA.

Based on the population PK model estimated in our study, BSA significantly affects the concentrations of linezolid in premature infants. BSA = 0.11, 0.13, and 0.15 m^2^ were taken into Monte Carlo stimulation to predict the linezolid concentration. Monte Carlo simulations were performed 1,000 times to investigate the exposure of linezolid in premature infants after four consecutive doses. As shown in Table 5, most dosage regimens reached the target range. The results indicated that the dosage regimens suggested by Monte Carlo stimulation were different from the labeled dosage. The prediction linezolid concentration versus time (Fig. 5) presented the change of concentration of 10 mg/kg q8h dosage regimen with different BSA. Table 6 shows the model-based prediction of linezolid trough concentrations (median) with the dose of 10 mg/kg q8h. Figure 6 shows the target attainment rates by minimum inhibitory concentration (MIC).

**TABLE 6:** Model-based prediction of linezolid trough concentrations (median) with the dose of 10 mg/kg q8h

Time after last dose (h)	Linezolid trough concentrations (μg/mL)		
	BSA = 0.11 m^2^	BSA = 0.13 m^2^	BSA = 0.15 m^2^
8	5.58	4.55	3.82
16	7.11	5.77	4.83
24	7.60	6.16	5.14
32	7.78	6.29	5.25
40	7.85	6.35	5.29
48	7.87	6.37	5.31

The optimal administration regimen for linezolid in premature neonates was 6 mg/kg q8h for BSA 0.11 m^2^, 7 mg/kg q8h for BSA 0.13 m^2^, and 9 mg/kg q8h for BSA 0.15 m^2^ with MIC ≤1 mg/L, 7 mg/kg q8h for BSA 0.11 m^2^, 8 mg/kg q8h for BSA 0.13 m^2^, and 10 mg/kg q8h for BSA 0.15 m^2^ with MIC = 2 mg/L.

## DISCUSSION

Although the clinical application of linezolid has increased progressively over the years, there are few pharmacokinetics data of linezolid in premature neonates. The present work is a prospective study to analyze and report on a premature neonatal PPK model, with dosage optimization of intravenous linezolid in premature neonates. The PPK model was established and validated to determine linezolid pharmacokinetics parameters in premature neonates and identify the impact of demographics and clinical factors on linezolid pharmacokinetics. Ultimately, the dosage was optimized in premature neonates with Monte Carlo simulation based on the final model.

The one-compartment model with first-order elimination described linezolid data well. The final model included BSA on clearance, and BSA on volume of distribution. A study indicated that PNA and WT were covariates in the linezolid PPK model of preterm infants, the median postnatal age (PNA) of preterm infants was 24 days (8–88 days), and BSA was not included as a tested covariate in the development of the PPK model in this study (5). All of the preterm neonates included in this study were diagnosed with late-onset sepsis, and linezolid was used as anti-infective therapy. The PNA was mostly in the range of 8–19 days during the treatment with linezolid. The differences in CL or V of the linezolid did not show a correlation with PNA, which is likely due to the small range of PNA. Crass et al. reported a one-compartment model with linear elimination in adult patients with renal impairment, and a covariate model building identified eGFR, BSA, and age as covariates of linezolid CL and BSA as a covariate of V (7). In premature neonates, BSA was found to be the main covariate influencing CL, possibly due to the maturity of the organs, especially the kidney. Neonatal kidney capacity is primarily related to renal maturity and hemodynamic stability. Preterm birth imposes immediate and potentially long-term alterations in kidney size and function. Kidney size individualized to the BSA is strongly correlated to kidney function (8).

In the final model, the median population estimate CL of linezolid in premature neonates was 0.098 L/h/kg, which is lower than the finding of a previous study of premature infants (0.13 L/h/kg)(5). This may be related to the following reasons: linezolid pharmacokinetics varies substantially in the first week of life depending on PNA, preterm infants aged >7 days had CL values that were approximately 3-fold greater than that aged <7 days (9), and the PNA values of infants in our study were smaller than those reported in the previous study of premature infants.

This is the first Monte Carlo simulation of intravenous linezolid in premature neonates. In adults, an AUC_0-24h_/MIC between 80 and 120 was the targeted pharmacodynamic parameters to ensure efficacy (10). Rao et al. suggested that an AUC_0-24_:MIC ratio of 80–100 is an appropriate efficacy threshold for children, showing that the clearance of linezolid in children was significantly faster than in adults (11). Currently, there is no recommended target range for neonates. Thibault et al. selected AUC_0-24_: MIC >80 and AUC_0-24_<300 as the target for premature infants (5). Numerous studies have reported a reasonably linear relationship between linezolid C_min_ and the AUC_0-24h_ (11). Considering the clinical difficulty of collecting multiple samples to reliably calculate the AUC_0-24_ :MIC ratio or T > MIC, evaluating the C_min_ based on the MIC remains a practical indicator of therapeutic efficacy and safety for linezolid. The determination of C_min_ is a common method for monitoring the toxicity of linezolid. Clinical studies have suggested target trough concentrations of 2–8 mg/L, 3.6–8.2 mg/L, or 2–7 mg/L for better efficacy and safety of linezolid (11). The expert consensus statement on the monitoring and individualization of linezolid therapeutics in 2022 recommended a linezolid trough concentration of 2–8 μg/mL, without distinguishing pediatrics or adults (2). Our simulations evaluated both C_min_ and AUC_0-24h_ , the target trough concentration range chosen in this study was 2–8 μg/mL, and the AUC_0-24h_ range chosen was 80–300 mg·h/L. In this study, linezolid was mainly used for neonatal late-onset sepsis and pneumonia, and most neonates were more than 7 days old on the first day of medication. The results of Monte Carlo simulations demonstrated that the currently recommended dose of linezolid for preterm neonate ≥7 days (10 mg/kg every 8 h) would lead to a high risk of overdosing for neonates. This is inconsistent with simulation results in previous studies of children aged 0–12 years, which demonstrated that the currently approved dosage (10 mg/kg every 8 h) would lead to a high risk of underdosing for children (12). This was consistent with results from previous studies showing that linezolid CL varied substantially in the pediatric population (13). The manufacturer’s study included 29 neonates in the pharmacokinetics section of the linezolid’s instruction, of which 20 patients were full-term neonates and only nine patients were premature neonates. The PNA of all nine preterm neonates was less than 7 days old. The drug instruction for linezolid does not specify the gestational age of the preterm infants as a reference for dosage regimens; meanwhile, there is also a lack of research support for dosing regimens with PNA greater than 7 days. The median postnatal age of the premature neonates included in our study was 13 days, which was different from that of the premature neonates included in the manufacturer’s study.

Monte Carlo simulations suggest that the optimal administration regimen for linezolid in premature neonates is 6 mg/kg q8h with BSA = 0.11 m^2^, 7 mg/kg q8h with BSA = 0.13 m^2^, and 9 mg/kg q8h with BSA = 0.15 m^2^. In cases with MICs of 1 mg/L, the optimal administration regimens would reach ＞ 90% PTAs. This is close to the simulation results of Thibault et al., where the recommended dosage of linezolid for preterm infants was 8 mg/kg q8h for MIC = 1 mg/L. However, when MIC = 2 mg/L, we recommend 7 mg/kg q8h with BSA = 0.11 m^2^, 8 mg/kg q8h with BSA = 0.13 m^2^, and 10 mg/kg q8h with BSA = 0.15 m^2^, a regimen that can achieve around 90% PTAs. The simulation results of Thibault et al. recommend that the dosage of linezolid for premature infants was 12 mg/kg q8h for MIC ≥2 mg/L, which could achieve >90% PTAs for MIC = 2 mg/L. However, according to the simulation results presented in this study, it was inferred that a linezolid dosage of 12 mg/kg q8h for neonates is prone to causing excess C_min_ and AUC_0-24_, which may increase the risk of toxicity.

We recommend that the initial dosages of linezolid for premature neonates be designed based on BSA and MIC. The dosing regimen is adjusted based on the steady-state C_min_, clinical efficacy, and adverse reactions. Bayesian approach and the population pharmacokinetic model can be used to guide the dose adjustment (11).

There are still some limitations in our study. (i) We only considered plasma concentrations of linezolid; there were some limitations on PK/PD targets and MIC values. (ii) This study had a limited sample size and required more concentrations other than C_min_. (iii) The population predictions of the full model were not so good; perhaps these were related to the great differences among neonate and premature populations, small dosages, or the fact that most of them were trough concentrations. We will use this PPK model to guide the individualized dosage regimens for linezolid and continue to collect concentration data to optimize this PPK model. (iv) This PPK model still needs clinical validation and the clinical intervention research of model-informed precision dosing. (v) The efficacy and safety of linezolid treatment were not evaluated.

In conclusion, we developed a population PK model of intravenous linezolid in premature neonates. BSA is the main factor affecting the CL and V_d_ of linezolid in premature neonates. The recommended dosage regimens based on the Monte Carlo simulations are much lower than the labeled dosage.

## MATERIALS AND METHODS

### Study design and patients

We retrospectively studied neonates hospitalized in neonatal intensive care (NICU) in Nanjing Medical University Affiliated Suzhou Hospital who underwent linezolid with TDM from November 2019 to November 2023. Premature infants treated with linezolid were included in the study. Those who had received linezolid for less than 2 days were excluded. Gestational age (GA), postmenstrual age (PMA), PNA, sex, birth weight, current weight, height, apgar scores, linezolid dosage, duration of linezolid treatment, pathogenic bacteria and MIC, serum concentrations of linezolid, blood test, C-reactive protein (CRP), procalcitonin (PCT), serum creatinine (Scr), and other biochemical tests were collected and further analyzed.

### Dosage regimen of linezolid

The empirical standard dosage regimens of linezolid injection (Zyvox; Pfizer Inc., New York, NY, USA) in infants were given based on linezolid manufacturer’s instructions: (1) 10 mg/kg, q12h for preterm neonates with a GA <34 weeks and a PNA <7 days and (2) 10 mg/kg, q8h for preterm neonates with a GA ≥34 weeks, and preterm neonates with a GA <34 weeks and a PNA ≥7 days. The course of the treatment was 7**–**14 days. The treatment may be discontinued early or extended appropriately based on clinical efficacy, adverse reactions, and other factors.

### Blood sampling and concentration determination

We measured the steady-state trough linezolid concentrations (after the fourth maintenance dose and 30 min prior to the next dose) and opportunistic concentrations (after the fourth maintenance dose). One or two blood samples (at least one trough concentration) were taken from each infant.

We took 1 mL of the whole blood, placed it in a coagulant/separation gel tube, and sent it to the medical laboratory for centrifugation within 2 h. The supernatant (serum) was separated and stored at −80°C, and the concentration of linezolid was determined within 3 days. Linezolid was extracted from the serum by protein precipitation, using acetonitrile containing an internal standard (levofloxacin). The blood concentration of linezolid was determined by the liquid chromatography-tandem mass spectrometry (LC-MS/MS) method, TRIPLE QUAD 4500MD mass spectrometry (AB SCIEX, United States), Jasper HPLC, and SB-AQ RRHD (50 × 3.0 mm, 1.8 m, Agilent, United States). The Analyst 1.6.1 data processing system is used for analysis. Chromatographic conditions: mobile phase A is 0.1% formic acid aqueous solution. The mobile phase B is 0.1% methanol formate solution; gradient elution; flow rate 0.4 ml/min; injection volume 2 µL; column temperature 45°C; 0–0.5 min, 10% B; 0.5–1.5 min, 10%–95% B; 1.5–2.0 min, 95% B; 2.0–2.2 min, 95%–10% B; and 2.2–3.0 min, 10%B. Mass spectrometry conditions were as follows: electrospray ionization element (ESI) and positive ion detection (MRM). Ion quantitative analysis reaction level included mass-to-charge ratio (M/Z) 338.6 → M/Z 296.2 (linezolid), cone voltage 60 V, collision energy 30 eV; (M/Z) 362.2 → (M/Z) 261.1 (internal standard solution, levofloxacin) cone voltage 65 V, and collision energy 35 eV. Quantifying of linezolid was validated over the 0.5–50 μg/mL concentration range with satisfactory accuracies (-0.59%–5.14%), intra-day precisions (≤3.45%), and inter-day precisions (≤6.99%). The mean extraction recoveries ranged from 97.63% to 102.30%, and the matrix effects were within the range of 99.59%**–**103.58%.

### Data collection

Demographic and clinical data (age, sex, weight, height, the Apgar score, linezolid dosage, linezolid medication days, infectious disease, pathogen, serum linezolid concentration, platelet count, and other data of examination) were collected and further analyzed.

### Pharmacokinetic analysis and model evaluation

The Phoenix NLME program (version 8.3, Pharsight, Mountain View, CA, USA) with the method of first-order conditional estimation-extended least square method (FOCE-ELS) was used to develop the linezolid population pharmacokinetic (PK) model. One- and two-compartment models with first-order elimination have been explored for the concentration-time data. The inter-individual variability is described by an exponential error model. To calculate the residual variability of PK parameters, the additive, proportional, and mixed (additive +proportional) models were tested. Model selection was based on the precision of parameter estimates (standard error), goodness-of-fit, and likelihood ratio test (−2LL).

The stepwise covariate modeling (SCM) approach was used to test the covariate model in this analysis; gender, gestational age, postnatal age, postmenstrual age, birth weight, current weight, body surface area (BSA), the apgar score, hemoglobin, platelet, alanine aminotransferase (ALT), aspartate aminotransferase (AST), total bilirubin (TBIL), serum albumin (ALB), serum creatinine, and clearance of creatinine (CLcr) were evaluated as the covariates. Correlation screening was conducted on all covariates, and if there was a correlation between covariates, only one of them was included.

BSA was estimated according to the formula of DuBois and DuBois (14).


(3) $$BSA(m^{2})=Weight(kg{)}^{0.425}\times height{\left(cm\right)}^{0.725}\times 0.07184$$

CLcr was calculated using the Schwartz formula, where K has a value of 0.33 for premature and neonates with low weight (WT) for gestational age (15). The Scr level was estimated using the Jafe method.


(4) $$CLcr[(ml/min)/1.73m^{2}]=\frac{K\times L}{SCR}$$

K：Correction factor，0.33 (Premature infants less than 1 year old)，L：body length(cm)，SCR：Blood creatinine measured by Jafé method (mg/dL) (15–17).

The physiological condition of the newborn changes greatly, the formula of physiological maturity is adopted for degrees (formulations 5 and 6) examines the effects of weight and age (18).


(5) $$CL_{p}=CL_{A}\times {\left(\frac{WT}{70}\right)}^{0.75}\times MF$$

(6) $$MF=\frac{PCA^{s}}{PCA^{s}+PCA_{50}^{s}}$$

In the above formula, CL_p_ is neonatal clearance rate, CL_A_ is adult clearance rate, MF is physiological maturity, PCA is postconceptional age, PCA_50_ is the PCA at which clearance reaches half its maximal value, and “s” is a sigmoidicity coefficient.

By comparing with the initial model, a drop >3.84 (*P* > 0.05) of the objective function value (OFV; −2LL) for forward addition and an increase of OFV >6.64 (*P* > 0.01) for backward elimination were the inclusion criteria for covariates.

The model evaluation was performed using statistical and graphical methods. GOF plots included scatterplots of population predictions (PRED) and individual predictions (IPRED) vs observed concentrations (DV), as well as conditional weighted residuals (CWRES) vs PRED and time after dose (TAD). A bootstrapping method by simulation of 1,000 subjects was used to assess the stability of the final model. The prediction-corrected visual predictive check (pc-VPC) was performed using 1,000 simulations to evaluate the predictive performance of the model.

### Monte Carlo simulation

To investigate whether the C_min_ and AUC_0-24h_ of different dosage regimens can reach the target range, Monte Carlo simulations were performed by referring to the labeled dosage and choosing several doses and dosing intervals. Based on the established PK model, BSA of 0.11, 0.13, and 0.15 m^2^ were stimulated. Monte Carlo simulations were performed for 1,000 individuals in each dosage regimen. The percentages of C_min_ and AUC_0-24h_ were evaluated. For all simulations, target C_min_ was between 2 µg/mL and 8 µg/mL (2), and target AUC_0-24h_ was between 80 and 300 mg·h/L (set MIC = 1) (5). For the assessment of efficacy, the probability of target attainment (PTA) of an AUC_0–24h_/MIC ratio threshold of 80 was calculated (5).

### Statistical analysis

The continuous variables are expressed as average. Binary and categorical data are expressed as counts. Differences between groups were assessed using the Wilcoxon test and Mann–Whitney U test. *P* value < 0.05 was considered significant. Data were analyzed by Graphpad Prism 9 (Graphpad company, USA) and SPSS 25 (IBM, USA) and presented in the form of statistical graphs using GraphPad Prism 9.
